# Uma Apresentação Rara de COVID-19 com Embolia Pulmonar

**DOI:** 10.36660/abc.20210350

**Published:** 2022-02-14

**Authors:** Özgenur Günçkan, Önder Öztürk, Veysel Atilla Ayyıldız, Volkan Bağlan, Münire Çakır, Ahmet Akkaya

**Affiliations:** 1 Department of Pulmonary Medicine Faculty of Medicine Süleyman Demirel University Isparta Turquia Department of Pulmonary Medicine, Faculty of Medicine, Süleyman Demirel University, Isparta – Turquia; 2 Department of Radiology Faculty of Medicine Süleyman Demirel University Isparta Turquia Department of Radiology, Faculty of Medicine, Süleyman Demirel University, Isparta – Turquia

**Keywords:** COVID-19, Embolia Pulmonar, Derrame Pleural

## Abstract

A doença de coronavírus 2019 (COVID-19) foi relatada em quase todos os países do mundo desde dezembro de 2019. A infecção por SARS-CoV-2 é frequentemente assintomática ou com sintomas leves, mas também pode levar à hipóxia, um estado hiperinflamatório e coagulopatia. Os parâmetros de coagulação anormais estão associados a complicações trombóticas, incluindo embolia pulmonar na COVID-19, mas pouco se sabe sobre os mecanismos. A semelhança dos sintomas iniciais de ambas as doenças também pode ser confusa, portanto, os médicos devem estar cientes do potencial para condições concomitantes. Apresentamos aqui um caso que não apresentava opacidades em vidro fosco nos pulmões, mas apresentava embolia pulmonar e derrame pleural em associação com infecção por COVID-19.

## Introdução

Um novo surto de doença causada por coronavírus (COVID-19) surgiu em Wuhan no final de dezembro de 2019 e se espalhou rapidamente para outros países, levando a uma pandemia devastadora. Os indivíduos infectados com a Síndrome Respiratória Aguda grave do Coronavírus 2 (SARS-CoV-2) foram admitidos nos hospitais com diferentes graus de gravidade da doença. A maioria deles é sintomática ou apresenta sintomas leves, enquanto alguns apresentam hipóxia, um estado hiperinflamatório e coagulopatia.^1 - 3^ A coagulopatia em COVID-19 foi demonstrada em autópsias, especialmente nas artérias pulmonares e capilares alveolares. Assim, embolias pulmonares (EP) concomitantes foram detectadas na tomografia computadorizada (TC) dos pacientes internados no hospital, mas a prevalência de EP em pacientes com COVID-19 permanece obscura.^1 , 2 , 4 - 7^ Apresentamos aqui um caso submetido a cirurgia devido a acidente no qual o diagnóstico foi dificultado pela coexistência de COVID-19 com EP e derrame pleural bilateral na hospitalização.

### Apresentação do caso

Uma mulher de 79 anos veio ao no nosso hospital com queixas de fraqueza, perda de apetite e falta de ar. A paciente apresentava histórico de queda do trator há um mês e havia sido submetida a cirurgia devido a fratura de úmero e fêmur. Ela tinha recebido alta hospitalar 12 dias antes da rehospitalização. Seu histórico familiar era normal e ela não tinha histórico de tabagismo e consumo de álcool.

### Exame físico na hospitalização

A paciente apresentava leve dispneia e estertores na base do pulmão esquerdo à auscultação. Ela apresentava temperatura de 36°C, frequência cardíaca de 78 batimentos/min e pressão arterial de 108/78 mmHg. A saturação de oxigênio medida por oxímetro de pulso foi de 92%.

### Achados laboratoriais

**Figura 1:**
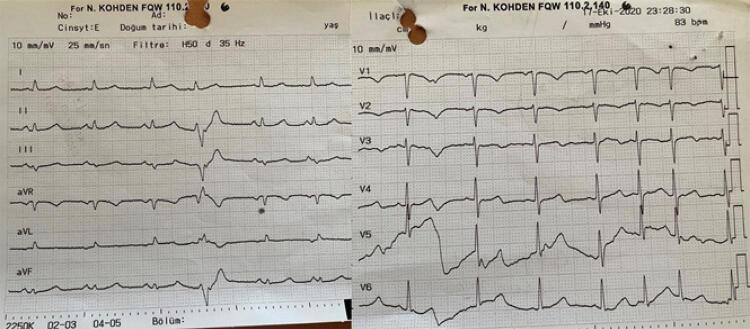
– O ECG do paciente era normal.

**Figura 2:**
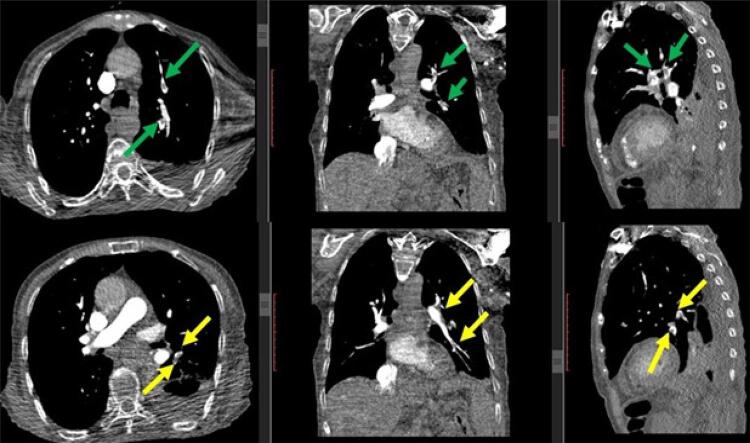
– Trombos hipodensos intraluminais no lobo proximal esquerdo superior e inferior (setas verdes e amarelas) nos ramos segmentares-subsegmentares da artéria pulmonar.

**Figura 3:**
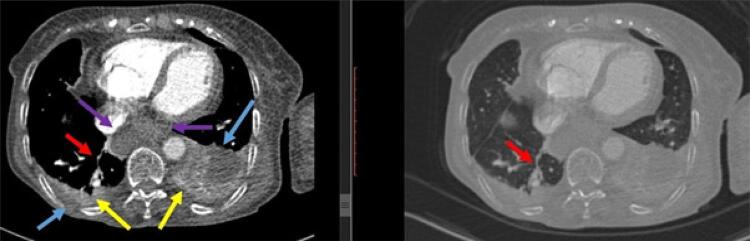
– Derrames pleurais bilaterais (setas azuis) e alterações atelectásicas compressivas adjacentes (setas amarelas), atelectasias subsegmentares (seta vermelha) e hérnia hiatal gastroesofágica tipo 1 (seta roxa).

As análises laboratoriais foram dignas de nota devido aos valores elevados de dímero D, proteína C reativa (PCR), troponina T e ferritina. A paciente também apresentou hipoxemia leve na gasometria arterial ( Tabela 1 ). O ECG da paciente foi normal. A angiografia pulmonar por tomografia computadorizada (APTC) mostrou embolia nos ramos periféricos segmentares de ambos os lobos inferiores do pulmão ( Figura 1 ), com derrame pleural bilateral ( Figura 2 ) e sequelas de alterações fibróticas e infiltrações ( Figura 3 ). Embora não houvesse padrão de vidro fosco no parênquima (achado atípico para COVID-19), um teste de reação em cadeia da polimerase (PCR) para COVID-19 foi realizado com esfregaço nasofaríngeo e considerado positivo na hospitalização.

**Tabela 1:** – Achados laboratoriais na hospitalização e após o tratamento

Parâmetros	Na hospitalização	Após o tratamento	Intervalo de referência
PCR (mg/dL)	64	17	0-5
Sedimentação (mm/h)	35	27	3-55 (>70 years old)
Procalcitonin (ng/mL)	0,177	0,054	<0,5
Leucócitos (x10^3^ cells/mm^3^)	5,2	2,9	5,2-12,4
Neutrófilos (x10^3^ cells/microL)	4,1	1,8	2,1-6,1
Linfócitos (x10^3^/microL)	0,6	0,6	1,3-3,5
Plaquetas (x10^3^/microL)	180	257	156-373
Hemoglobina (g/dL)	10,3	10,9	13,6-17,2
Hematócrito (%)	30,9	33,2	39,5-50,3
D-dímero (ng/L)	2454	858	69-243
TP (sec)	16,7	17,7	9,4-12,5
PTTa (sec)	27,5	32	25,4-38,4
TT (sec)	23,7	-	15,8-24,9
INR INR	1,42	1,51	0,8-1,1
Fibrinogênio (mg/dL)	301	419	200-393
Ferritina (ng/dL)	1057	1022	4,63-204
Troponina T (ng/mL)	0,065	0,024	0-0,014
LDH (U/L)	321	349	0-247
ALT (U/L)	6	11	0-34
AST (U/L)	18	29	0-31
Creatina (mg/dL)	0,31	0,28	0,66-1,29
Proteína (g/dL)	5,45	5,64	6,6-8,3
Albumina (g/dL)	2,8	2,9	3,5-5,2
Na (mmol/L)	130	138	136-146
K (mmol/L)	3,77	3,97	3,3-5,1
Ca (mg/dL)	7,67	8,37	8,8-10,6
Ca corrigido (mg/dL)	8,63	9,17	9,2-9,64

*PCR: proteína C reativa; TP: tempo de protrombina; PTTa: tempo de tromboplastina parcial ativada; TT: tempo de trombina; INR: International normalized ratio; LDH: lactato desidrogenase; ALT: alanina aminotransferase; AST: Aspartato aminotransferase; Na: sódio; K: potássio; Ca: cálcio.*

### Diagnóstico final e tratamento

O diagnóstico final da paciente foi infecção por COVID-19 com EP e derrame pleural bilateral. A paciente foi transferida para o serviço de pacientes com resultado positivo para COVID e tratada com favipiravir (2x1600 mg/dia no primeiro dia e 2x600 mg/dia nos quatro dias seguintes), moxifloxacina 1x400 mg/dia e heparina de baixo peso molecular (HBPM) 2x0,6 IU. Dez dias depois, ela teve alta hospitalar sem necessidade de oxigênio suplementar. Foi prescrita heparina de baixo peso molecular por um mês e o tratamento foi continuado posteriormente com anticoagulantes orais.

## Discussão

No estudo atual, a paciente apresentou infecção por COVID-19 e EP concomitante, com derrame pleural. As queixas no momento da hospitalização eram fraqueza, perda de apetite e falta de ar, que eram esperadas durante a infecção por COVID-19, mas não na EP e derrames pleurais, exceto pela queixa de dispneia.^8 , 9^ Em relação aos achados laboratoriais, os níveis de ferritina e PCR, troponina e dímero D estavam elevados, como observado em pacientes com COVID-19 em uma meta-análise.^10^ Uma das características mais típicas das infecções por COVID-19 são imagens periféricas/subpleurais bilaterais em padrão de vidro fosco (97,6%) na TC de tórax, enquanto a consolidação, espessamento do septo interlobular e padrão de pavimentação em mosaico são vistos em 63,9%, 62,7% e 36,1% dos pacientes, respectivamente.^9^ Entretanto, o derrame pleural e o derrame pericárdico são vistos entre 3% a 28% dos pacientes.^9 , 11^ Foi relatado que a distribuição dos achados de imagem varia de acordo com a idade. Verificou-se que a opacidade em vidro fosco (GGO, do inglês *ground-glass opacity* ) foi observada principalmente em indivíduos mais jovens (<50 anos) (77%), e as consolidações com padrão de pneumonia em organização e consolidação pura foram encontradas em pessoas com idades mais avançadas (45%).^12^ Embora derrames pleurais tenham sido encontrados mais comumente em pacientes idosos, ainda não está estabelecido se a idade é um possível fator de risco para o desenvolvimento de derrames pleurais em pacientes com COVID-19. Além disso, a importância dos derrames pleurais na pneumonia por COVID-19 ainda não foi bem avaliada devido à raridade da doença, limitada a relatos/séries de casos.^7 , 13^

Embora um aumento no estado de coagulação tenha sido relatado em pacientes infectados com SARS-CoV-2 em comparação com controles saudáveis, há publicações limitadas sobre a prevalência ou incidência de embolia pulmonar.^14 , 15^ Assim, será um passo valioso realizar tomografias computadorizadas de tórax contrastadas para pacientes com pneumonia por COVID-19 que apresentam início súbito de dispneia ou aqueles com níveis elevados de dímero D para excluir embolia pulmonar, porque a mesma pode ser uma complicação da pneumonia viral.^16^ A anormalidade laboratorial mais comum na coagulopatia por COVID-19 são os níveis elevados de dímero-D, que refletem a ativação da cascata de coagulação, como visto em nossa paciente.^5^ A capacidade discriminatória do dímero-D está substancialmente reduzida em comparação com a população em geral, e a evidência de altos níveis séricos de D-dímero isoladamente não pode ser considerada para fins diagnósticos.^4^ Portanto, os médicos devem considerar todos os pacientes com COVID-19 em risco de tromboembolismo venoso, especialmente na presença de hospitalização tardia após o início dos sintomas, perfil de biomarcadores séricos de alto risco e evidência ecocardiográfica de disfunção ventricular direita e hipertensão pulmonar, todos os quais devem alertar os médicos para a presença de EP.^4^

Em conclusão, níveis elevados de D-dímero (acima de 1743 ng/mL) podem estar relacionados ao diagnóstico de EP durante a pandemia de COVID-19. Devemos estar cientes da possibilidade de ocorrência concomitante de EP e COVID-19, principalmente em pacientes com sintomas como fraqueza e perda de apetite, que não podem ser explicados pela EP isoladamente.
