# Supplementary material for: Uma Apresentação Rara de COVID-19 com Embolia Pulmonar
Source: Arq Bras Cardiol. 2022 Feb 14;118(2):525–9. [Article in Portuguese] doi: 10.36660/abc.20210350 (PMC8856685; doi:10.36660/abc.20210350)
Supplement: Translation

# Translation: A Rare Presentation of COVID-19 with Pulmonary Embolism

**Authors:** Özgenur Günçkan, Önder Öztürk, Veysel Atilla Ayyıldız, Volkan Bağlan, Münire Çakır, Ahmet Akkaya

**Affiliations:** 1 Turkey Department of Pulmonary Medicine, Faculty of Medicine, Süleyman Demirel University, Isparta – Turkey; 2 Turkey Department of Radiology, Faculty of Medicine, Süleyman Demirel University,Isparta – Turkey

**Keywords:** COVID-19, Pulmonary Embolism, Pleural Effusion

## Abstract

Coronavirus disease 2019 (COVID-19) has been reported in almost every country in the world since December 2019. Infection with SARS-CoV-2 is often asymptomatic or with mild symptoms, but it may also lead to hypoxia, a hyperinflammatory state, and coagulopathy. The abnormal coagulation parameters are associated with thrombotic complications, including pulmonary embolism in COVID-19, but little is known about the mechanisms. The similarity of initial symptoms of both diseases can also be confusing, therefore the physicians should be aware of the potential for concurrent conditions. Herein, we present a case who did not have ground-glass opacities in the lungs, yet presented with pulmonary embolism and pleural effusions in association with COVID-19 infection.

## Introduction

A novel coronavirus disease (COVID-19) outbreak emerged in Wuhan in late December 2019 and spread rapidly to other countries, leading to a devastating pandemic. The individuals infected with severe acute respiratory syndrome coronavirus 2 (SARS-CoV-2) have been admitted to the hospitals with different degrees of disease severity. Most of them are symptomatic or show mild symptoms, while some of them have hypoxia, a hyperinflammatory state, and coagulopathy_._^1 - 3^ The coagulopathy in COVID-19 has been demonstrated in autopsies, especially in the pulmonary arteries and alveolar capillaries. Thus, concomitant pulmonary embolisms (PE) have been detected on the computed tomography (CT) scans of the patients admitted to the hospital, but the prevalence of PE in patients with COVID-19 remains unclear.^1 , 2 , 4 - 7^ Herein, we present a case who was operated on due to an accident and the diagnosis was complicated by the coexistence of COVID-19 with PE and bilateral pleural effusions on admission.

### Case presentation

A 79-year-old woman presented to our hospital with complaints of weakness, loss of appetite, and shortness of breath. The patient had a history of falling from the tractor one month before and had been operated on for fractures in the humerus and femur. She had been discharged from the hospital 12 days before readmission. Her family history was unremarkable and she had no history of smoking and alcohol use.

### Physical examination upon admission

The patient had mild dyspnea and rales in the left lung base on auscultation. She had a temperature of 36°C, a heart rate of 78 beats/min, and blood pressure of 108/78 mmHg. The oxygen saturation measured by a pulse oximeter was 92%.

### Laboratory findings

The laboratory analyses were noteworthy due to elevated levels of D-dimer, C-reactive protein (CRP), troponin-T, and ferritin. There was also mild hypoxemia on arterial blood gas analysis ( Table 1 ). The patient’s ECG was normal ( Figure 1 ). Computed tomography pulmonary angiography (CTPA) scan showed embolism at the peripheral segmental branches of both lower pulmonary lobes ( Figure 1 ), with bilateral pleural effusions ( Figure 2 ), and fibrotic changes and infiltrations as sequelae ( Figure 3 ). Although there was no ground glass opacity in the parenchyma (atypical findings for COVID-19), a polymerase chain reaction (PCR) test for COVID-19 was performed on the nasopharyngeal smear and was found positive at hospitalization.

**Table 1:** – Laboratory findings on hospital admission and after treatment.

Parameters	On hospital admission	After treatment	Reference range
CRP (mg/dL)	64	17	0-5
Sedimentation (mm/h)	35	27	3-55 (>70 years old)
Procalcitonin (ng/mL)	0.177	0.054	<0.5
WBCs (x10^3^cells/mm^3^)	5.2	2.9	5.2-12.4
Neutrophils (x10^3^cells/microL)	4.1	1.8	2.1-6.1
Lymphocytes (x10^3^/microL)	0.6	0.6	1.3-3.5
Platelets (x10^3^/microL)	180	257	156-373
Hemoglobin (g/dL)	10.3	10.9	13.6-17.2
Hematocrit (%)	30.9	33.2	39.5-50.3
D-dimer (ng/L)	2454	858	69-243
PT (sec)	16.7	17.7	9.4-12.5
aPTT (sec)	27.5	32	25.4-38.4
TT (sec)	23.7	-	15.8-24.9
INR INR	1.42	1.51	0.8-1.1
Fibrinogen (mg/dL)	301	419	200-393
Ferritin (ng/dL)	1057	1022	4.63-204
Troponin T (ng/mL)	0.065	0.024	0-0.014
LDH (U/L)	321	349	0-247
ALT (U/L)	6	11	0-34
AST (U/L)	18	29	0-31
Creatine (mg/dL)	0.31	0.28	0.66-1.29
Protein (g/dL)	5.45	5.64	6.6-8.3
Albumin (g/dL)	2.8	2.9	3.5-5.2
Na (mmol/L)	130	138	136-146
K (mmol/L)	3.77	3.97	3.3-5.1
Ca (mg/dL)	7.67	8.37	8.8-10.6
Corrected Ca (mg/dL)	8.63	9.17	9.2-9.64

*CRP: C-reactive protein; WBCs: White blood cells; PT: Prothrombin time; aPTT: Activated partial thromboplastin time; TT: Thrombin time; INR: International normalized ratio; LDH: Lactate dehydrogenase; ALT: Alanine aminotransferase; AST: Aspartate aminotransferase; Na: Sodium; K: Potassium; Ca: Calcium.*

**Figure 1:** – The ECG of the patient was normal.

**Figure 2:** – Intraluminal hypodense thrombi in the proximal left upper and lower lobes (green and yellow arrows) in the segmental-subsegmental pulmonary artery branches.

**Figure 3:** – Bilateral pleural effusions (blue arrows) and adjacent compressive atelectatic changes (yellow arrows), subsegmental atelectatis (red arrow), and type 1 gastroesophageal hiatal hernia (purple arrow).

### Final diagnosis and treatment

The final diagnosis of the patient was COVID-19 infection with PE and bilateral pleural effusions. The patient was transferred to the COVID-positive service and treated with favipiravir (2x1600 mg/day on the first day and 2x600 mg/day for the next four days), moxifloxacin 1x400 mg/day and 2x0.6 IU low-molecular-weight heparin (LMWH). Ten days later she was discharged from the hospital without the need for oxygen supplementation. The patient was prescribed low molecular weight heparin for one month and the treatment was subsequently continued with oral anticoagulants.

## Discussion

In the current study, the patient presented with COVID-19 infection and concomitant PE, with pleural effusions. The complaints at the time of hospital admission were weakness, loss of appetite, and shortness of breath, which were expected to occur in a case of COVID-19 infection, but not expected in PE and pleural effusions, except for the dyspnea complaint.^8 , 9^ Regarding the laboratory findings, the ferritin and CRP, troponin, and D-dimer levels were found to be elevated, as observed in patients with COVID-19 in a meta-analysis.^10^ One of the most characteristic features of COVID-19 infections is peripheral/subpleural bilateral ground glass opacities (97,6%) on chest CT, whereas consolidation, interlobular septal thickening, crazy-paving pattern are seen in 63.9%, 62,7%, and 36,1% of the patients, respectively.^9^ However, pleural effusion and pericardial effusion are seen between 3% to 28% of the patients.^9 , 11^ It was reported that the distribution of imaging findings varies according to age. It was found that the ground-glass opacity (GGO) was mostly seen at younger ages (< 50 years old) (77%), and the consolidations with an organizing pneumonia pattern and pure consolidation were found at older ages (45%).^12^ Although pleural effusions were more commonly found in elderly patients, it is uncertain whether age is a possible risk factor for the development of pleural effusion in COVID-19 patients. Furthermore, the significance of pleural effusions in COVID-19 pneumonia has not been well assessed due to the rarity of the disease, limited to case reports/series.^7 , 13^

Although an increased coagulation state has been reported in patients infected with SARS-CoV-2 when compared to healthy controls, there are limited publications on the prevalence or incidence of pulmonary embolism.^14 , 15^ Thus, it will be a valuable step to perform contrasted chest CT scans in patients with COVID-19 pneumonia who present with sudden onset of dyspnea or those with elevated D-dimer levels to exclude pulmonary embolism, because the latter may be a complication of viral pneumonia.^16^ The most common laboratory abnormality in COVID-19 coagulopathy is elevated D-dimer levels, which reflect the activation of the coagulation cascade, as seen in our patient.^5^ The discriminative ability of D-dimer is substantially reduced when compared to the general population, and the evidence of high D-dimer serum levels alone cannot be considered for the diagnostic purposes.^4^ Therefore, the clinicians should consider all COVID-19

patients to be at risk of venous thromboembolism, especially in the presence of late hospitalization after symptom onset, high-risk serum biomarker profile, and echocardiographic evidence of right ventricular dysfunction and pulmonary hypertension, all of which must alert the clinicians for the presence of PE.^4^

In conclusion, high levels of D-dimer (higher than 1743 ng/mL) may be related to a diagnosis of PE during the COVID-19 pandemia. We should be aware of the possibility of overlapping PE and COVID-19, especially in patients with symptoms such as weakness and loss of appetite, which cannot be explained by PE alone.
